# α‐Hederin induces paraptosis by targeting GPCRs to activate Ca^2+^/MAPK signaling pathway in colorectal cancer

**DOI:** 10.1002/cam4.7202

**Published:** 2024-04-25

**Authors:** Xiwu Rao, Ziwen Li, Qinchang Zhang, Yueyang Lai, Jianrong Liu, Liu Li, Haibo Cheng, Weixing Shen, Dongdong Sun

**Affiliations:** ^1^ The First Clinical Medical College of Nanjing University of Chinese Medicine Collaborative Innovation Center of Jiangsu Province of Cancer Prevention and Treatment of Chinese Medicine Nanjing China; ^2^ Department of Oncology The First Affiliated Hospital of Guangzhou University of Chinese Medicine Guangzhou China; ^3^ Guangzhou University of Chinese Medicine Guangzhou China; ^4^ Postdoctoral Research Station of Guangzhou University of Chinese Medicine Guangzhou China; ^5^ Department of Infectious Disease Nanjing Hospital of Chinese Medicine Affiliated to Nanjing University of Chinese Medicine Nanjing China

**Keywords:** calcium, colorectal cancer, MAPK, paraptosis, α‐Hederin

## Abstract

**Background:**

Non‐apoptotic cell death is presently emerging as a potential direction to overcome the apoptosis resistance of cancer cells. In the current study, a natural plant agent α‐hederin (α‐hed) induces caspase‐independent paraptotic modes of cell death.

**Purpose:**

The present study is aimed to investigate the role of α‐hed induces paraptosis and the associated mechanism of it.

**Methods:**

The cell proliferation was detected by CCK‐8. The cytoplasm organelles were observed under electron microscope. Calcium (Ca^2+^) level was detected by flow cytometry. Swiss Target Prediction tool analyzed the potential molecule targets of α‐hed. Molecular docking methods were used to evaluate binding abilities of α‐hed with targets. The expressions of genes and proteins were analyzed by RT‐qPCR, western blotting, immunofluorescence, and immunohistochemistry. Xenograft models in nude mice were established to evaluate the anticancer effects in vivo.

**Results:**

α‐hed exerted significant cytotoxicity against a panel of CRC cell lines by inhibiting proliferation. Besides, it induced cytoplasmic vacuolation in all CRC cells. Electron microscopy images showed the aberrant dilation of endoplasmic reticulum and mitochondria. Both mRNA and protein expressions of Alg‐2 interacting proteinX (Alix), the marker of paraptosis, were inhibited by α‐hed. Besides, both Swiss prediction and molecular docking showed that the structure of α‐hed could tightly target to GPCRs. GPCRs were reported to activate the phospholipase C (PLC)‐β3/ inositol 1,4,5‐trisphosphate receptor (IP3R)/ Ca^2+^/ protein kinase C alpha (PKCα) pathway, and we then found all proteins and mRNA expressions of PLCβ3, IP3R, and PKCα were increased by α‐hed. After blocking the GPCR signaling, α‐hed could not elevate Ca^2+^ level and showed less CRC cell cytotoxicity. MAPK cascade is the symbol of paraptosis, and we then demonstrated that α‐hed activated MAPK cascade by elevating Ca^2+^ flux. Since non‐apoptotic cell death is presently emerging as a potential direction to overcome chemo‐drug resistance, we then found α‐hed also induced paraptosis in 5‐fluorouracil‐resistant (5‐FU‐R) CRC cells, and it reduced the growth of 5‐FU‐R CRC xenografts.

**Conclusions:**

Collectively, our findings proved α‐hed as a promising candidate for inducing non‐apoptotic cell death, paraptosis. It may overcome the resistance of apoptotic‐based chemo‐resistance in CRC.

## 1 INTRODUCTION

Colorectal cancer (CRC) incidence and mortality is rapidly growing worldwide.^1^ The advancements made in the treatment of CRC, mainly include surgery and chemotherapy. 5‐fluorouracil (5‐FU)‐based chemotherapeutic regimens are the conventional treatments for CRC, but it is usually not highly effective against metastatic colorectal cancer (mCRC), and resistance of chemotherapy will accelerate the progression of CRC. Thus, it is crucial to discover novel strategies for CRC treatment.

Cell death is a fundamental cellular process. In certain circumstances, cells regulate their death in a programmed dependent way. Apoptosis is the classical form of programmed cell death.^2^ Inhibition or evasion of apoptosis is partially characterized by CRC,^3^ which in turn endows tumors advantage for survival and contributes to anti‐cancer therapy resistance.^4^ Therefore, characterization of non‐apoptotic cell death may suggest strategies that could offer alternatives to anti‐cancer therapeutic approaches.

Paraptosis is a distinct form of non‐apoptotic cell death.^5^, ^6^, ^7^, ^8^ The feature of paraptosis is accompanied by massive cytoplasmic vacuolation,^9^ alterations in organelle structure, and enlargement of both the endoplasmic reticulum (ER) and mitochondria.^10^ The regulatory pathways of paraptosis encompass mitogen‐activated protein kinases (MAPKs) and its modulation is inversely associated with the activity of Alg‐2 interacting proteinX (Alix).^11^ Paraptosis was reported to occur in various cancer cells that were treated with natural anti‐cancer agents.^5^ Curcuminoid B63 has been shown to elicit paraptosis in gastric cancer cells through ROS‐induced ER stress coupled with MAPK pathway activation.^12^ Similarly, Jolkinolide B incites both paraptosis and apoptosis in bladder cancer cells through ROS‐driven ER stress mechanisms.^8^ Furthermore, in non‐small cell lung cancer cells, celastrol has been identified as an inducer of paraptosis to overcome resistance to afatinib.^13^

α‐Hederin (α‐hed) is a chemical compounds that can be extracted by *Akebia trifoliata* (Thunb.) Koidz plants. It has gained attention for its anti‐cancer effects in recent years.^14^, ^15^ The present study shows that α‐hed induced paraptosis‐like cell death, the proparaptotic activity of α‐hed in CRC cells was mediated through targeting G‐protein‐coupled receptors (GPCRs) to activate phospholipase C (PLC)‐β3, PLCβ3 produced signals to stimulate calcium (Ca^2+^) signaling by activation of inositol 1,4,5‐trisphosphate receptor (IP3R), which subsequently activated protein kinase C alpha (PKCα) and led to the MAPK activation. Interestingly, α‐hed was effective against chemotherapy‐resistant CRC both in vivo and in vitro. Our results suggest that α‐hed may provide an alternative therapeutic strategy for CRC.

## 2 MATERIALS AND METHODS

### 2.1 Cell lines and reagents

CRC cell lines, including LoVo, HT29, and HCT‐116, were sourced from the American Type Culture Collection. To establish stable 5‐FU resistant CRC cells, HT‐29 cell line were treated with gradually increasing concentration of 5‐FU (from 10 to 300 μM initially), cells were passaged three times at each 5‐FU concentration of 10, 20, 40, 80, 150, 300 μM. We assessed 5‐FU resistance at each dose by calculating the IC50 using CCK8 assays. These lines were propagated in DMEM medium from KeyGen Biotechn, Nanjing, China, which was enriched with 10% fetal bovine serum and 1% penicillin–streptomycin, both acquired from Gibco, Eggenstein, Germany. Meanwhile, α‐hederin was procured from Chengdu Herbpurify CO., LTD (purity>98%), the chemical structure of α‐hederin is shown in Figure S1. α‐Hederin was initially dissolved in DMSO before being further diluted using the aforementioned DMEM medium.

### 2.2 Cell viability assay

For CRC cell viability analysis, 5 × 10^3^ cells were dispensed into each compartment of a 96‐well format. Subsequently, the cells were exposed to varying doses of α‐hed ranging from 0 to 56 μM. In another setting, a consistent concentration of 24 μM α‐hed was administered for distinct time intervals: 0, 6, 12, 24, 48, and 72 h. Post‐treatment, the CCK‐8 assay from Beyotime, Nantong, China, was utilized to determine cell viability. Absorbance readings were procured at a wavelength of 450 nm using instrumentation from Tecan Group Ltd., Männedorf, Switzerland.

### 2.3 Transmission electron microscopy

Exposure of HT‐29 cells to DMSO served as the vehicle control, whereas another set experienced 16 μM α‐hed intervention for a duration of 24 h. Subsequent procedures involved fixing these cells in 2.5% glutaraldehyde, maintained at 4°C over a 12‐h period. This was followed by post‐fixation using 1% OsO₄ for an additional hour, staining processes with uranyl acetate, and a dehydration step. The final embedding was done using the Poly/Bed 812 resin, sourced from Pelco. Detailed cellular observations were carried out with an electron microscope, specifically the FEI Tecnai G2 from Thermo Scientific, USA.

### 2.4 Western blotting

Cell lysates underwent preparation, and their protein concentrations were quantified using the BCA method, a product of Thermo Scientific, USA. A 12% SDS‐PAGE allowed the separation of these proteins, followed by their transfer to membranes made of poly‐vinylidene difluoride. Once transferred, these membranes underwent a blocking phase for a span of 2 h using 10% bovine serum albumin in TBST that was freshly mixed. Post‐blocking, the membranes were exposed to designated primary antibodies for a period of 12 h. For visualization purposes, membranes were treated with HRP‐conjugated secondary antibodies sourced from Beyotime, Nantong, China, alongside the ECL substrate from Thermo Scientific, USA. The antibodies used for the experiment included: Alix (CST, 2171S, 1:1000 dilution), ERK (CST, 137F5), p‐ERK (CST, 4370S, 1:1000), JNK (proteintech, 66210‐1‐Ig, 1:3000), p‐JNK (proteintech, 80024‐1‐RR, 1:2000), p38 (CST, 8690S, 1:1000), p‐p38 (CST, 4511S, 1:1000), and GAPDH (CST, 5174S, 1:1000).

### 2.5 Reverse transcription and quantitative real‐time PCR

For the synthesis of cDNA, total RNA underwent reverse transcription utilizing the PrimeScript RT Reagent Kit, a product from Vazyme, Nanjing, China. The quantification process for Alix, PLCβ3, IP3R, and PKC was carried out with their expression levels adjusted based on GAPDH. The primers specified in subsequent sections were employed for the RT‐qPCR execution (Table 1).

**TABLE 1:** List of primers.

Genes	Forward	Reverse
Alix	CTGGAAGGATGCTTTCGATAAAGG	AGGCTGCACAATTGAACAACAC
PLCβ3	TGGAGCTGGACGTGTGGAAGG	GAAGGCAGTCTCGGCAATGGC
IP3R	TGCGAATGAATCCGCCATTGTCAC	AACTGCATCCTTTGGTTCAAGCCG
PKC	GCCTATG GCGTCCTGTTGTATG	GAAACAGCCTCCTTGGACAAGG
GAPDH	CAAGTTCAACGGCACAGTCAAG	ATACTCAGCACCAGCATCACC

### 2.6 Swiss target prediction assay

Swiss Target Prediction tool (http://www.swisstargetprediction.ch) analyzed the potential molecule targets of α‐hed.

### 2.7 Molecular docking of α‐hed to the GPCRs structural model

Molecular docking methods were used to evaluate binding abilities of α‐hed with GPCRs. The 2D structure of α‐hed was downloaded from PubChem database, and then transformed into 3D structure with mimimum structural energy by ChemBio3D program. 3D structures of the GPCRs were retrieved from PDB database, and dehydrated by PyMOL 2.5.2. Then, AutoDockTools 1.5.6 performed molecular docking of α‐hed with GPCRs. PyMOL 2.5.2 was used to visualize the binding modes of α‐hed and GPCRs.

### 2.8 CRC xenografts

The Institutional and Local Committee on the Care and Use of Animals at Nanjing University of Chinese Medicine granted permission for all described experiments (Ethical approval number, 202110A025). Nude male mice, 5 weeks old and weighing between 18 and 22 g, were procured from Vital River Laboratories, Beijing, China. These mice (*N* = 30) were distributed randomly across five distinct groups. Each mouse received a subcutaneous injection on their right flank with HT‐29 cells, where the injection volume was 100 μL PBS containing 1 × 10^6^ cells. Following a period of 14 days post‐injection, when tumors approached an approximate volume of 100 mm^3^, a regimen of treatments commenced. The mice underwent intraperitoneal injections either with 5‐FU at a dose of 25 mg/kg or α‐hed at dosages of 1.5, 1, or 0.5 mg/kg, administered biweekly. This treatment spanned 21 days. On Days 7, 14, 21, 28, and 35, tumor dimensions, including length and width, were recorded. The volume was then computed using the formula *V* = 0.5*l*w^2^. Concluding the experiments on the 35th day, the mice underwent euthanasia, post which tumor tissues were extracted and subsequently weighed.

### 2.9 Tissue staining

Tumor samples underwent fixation in a 10% formalin solution prior to being embedded in paraffin. Subsequent sectioning yielded slices with a thickness of 3 μm. For the purpose of observing histopathological changes, staining was conducted using hematoxylin and eosin (H&E). The immunohistochemical (IHC) methodology implemented included the use of primary antibodies targeting Alix and ki‐67, both at a dilution ratio of 1:200. Post incubation with the respective secondary antibody, visualization of positive staining was achieved through the application of diaminobenzidine (DAB). Moreover, the TUNEL assay, relying on a colorimetric approach, was executed in accordance with guidelines provided by Beyotime.

### 2.10 Statistical analysis

For the purposes of data analysis, the GraphPad Prism version 8 platform was employed. Utilizing one‐way ANOVA, variations of significance were assessed. A threshold of *p* < 0.05 served as the criterion for deeming differences to hold statistical significance.

## 3 RESULTS

### 3.1 α‐Hed inhibits cell viability and induces cytoplasmic vacuolation in CRC cells

**FIGURE 1:**
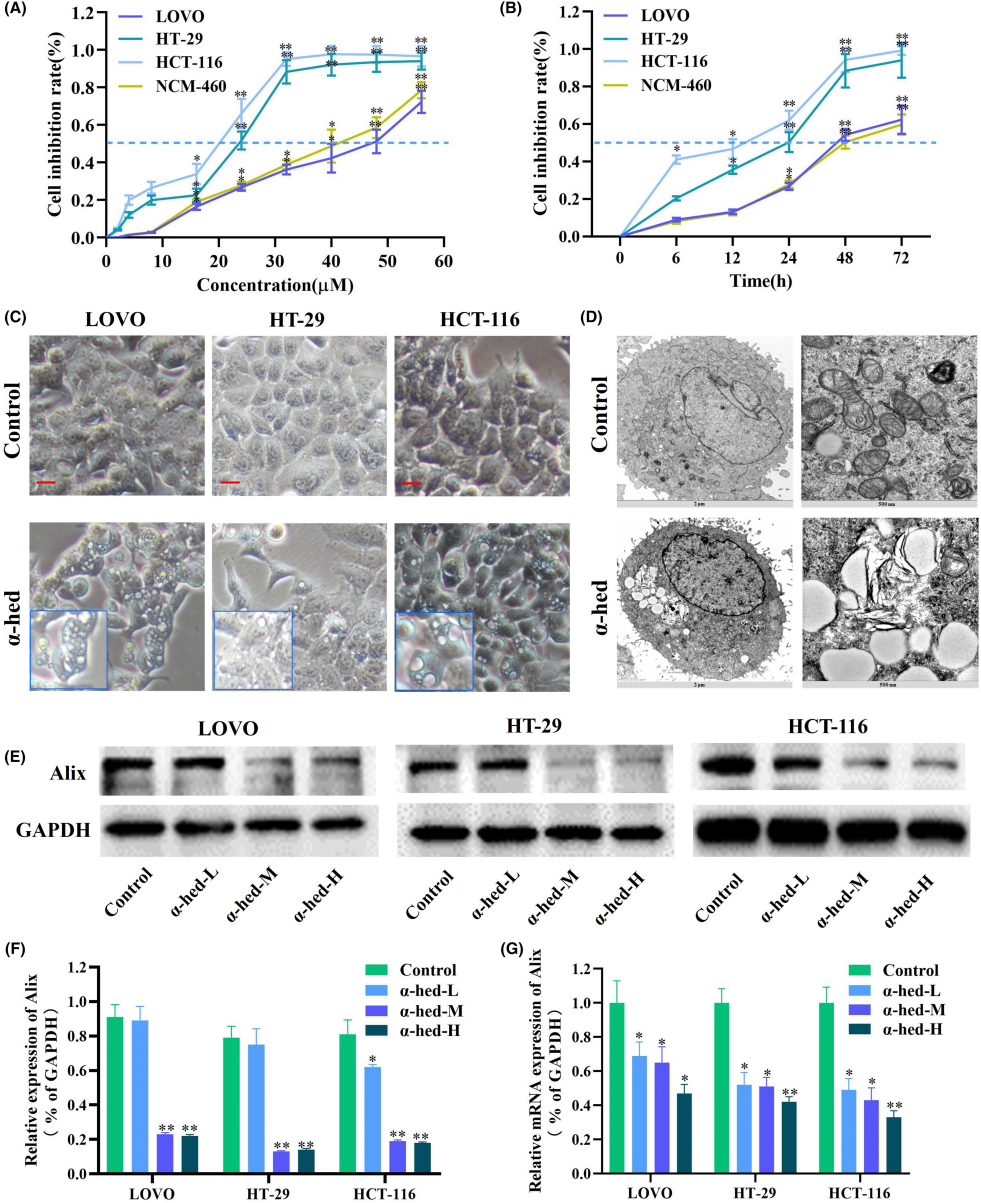
α‐Hed inhibits cell viability and induces cytoplasmic vacuolation in CRC cells. (A) CRC cell lines (LOVO, HT‐29, HCT‐116) and human colon epithelial cell (NCM‐460) underwent treatment across varying α‐hed concentrations (ranging from 0 to 56 μM) over a span of 24 h, followed by the assessment of cell viability through the CCK‐8 assay method. (B) CRC cell lines (LOVO, HT‐29, HCT‐116) and human colon epithelial cell (NCM‐460) were exposed to a consistent α‐hed concentration of 24 μM over varied time intervals (0, 6, 12, 24, 48, 72 h). Subsequent cell viability was evaluated using the CCK‐8 assay. (C) Phase contrast microscopy observations were made on LOVO, HT‐29, and HCT‐116 cells after 24 h of exposure to α‐hed at concentrations of 24, 12, and 10 μM respectively, [scale bar =50 μm]. (D) Following a 24 h treatment with 16 μM α‐hed, HT‐29 cell morphological changes were inspected using electron microscopy, revealing mitochondrial and ER swelling post‐treatment, [Magnification: either ×10,000 or 30,000, scale bars at 2 μm or 500 nm]. (E, F) Western blot investigations were conducted for Alix expression. For LOVO cells, the treatment was categorized into three dosages: low at 12 μM (α‐hed‐L), medium at 24 μM (α‐hed‐M), and high at 48 μM (α‐hed‐H). HT‐29 cells were exposed to 6, 12, and 24 μM, while HCT‐116 cells had treatments of 5, 10, and 20 μM for the same categories. GAPDH served as the loading reference, with densitometric quantifications performed via ImageJ. (G) Alix's expression levels were assessed using RT‐qPCR. The categorizations for LOVO, HT‐29, and HCT‐116 remain aligned with the prior description. GAPDH functioned as the loading control. Significant differences are denoted as *p < 0.05, **p < 0.01 in comparison with the control group.

In assessing the potential anti‐tumor capabilities of α‐hed against CRC cells, three distinct CRC cell strains (LOVO, HT‐29, and HCT‐116) and one normal human colon epithelial cell (NCM‐460) underwent treatment with varying α‐hed concentrations for a 24‐h period, followed by viability testing using the CCK‐8 method. A marked inhibition in cell proliferation was observed across all strains, manifesting in a dose‐responsive manner. The corresponding IC50 values were approximately 48 μM for LOVO, 23 μM for HT‐29, and 19 μM for HCT‐116, 40 μM for NCM‐460, as indicated in Figure 1A. When exposed to 24 μM of α‐hed, a time‐sensitive suppression of proliferation was apparent in each cell strain. The viability was reduced by half at around 14 h for HCT‐116, 24 h for HT‐29, 46 h for LOVO, and 48 h for NCM‐460 (Figure 1B).

Interestingly, cytoplasmic vacuolation was observed in LOVO, HT‐29, and HCT‐116 cells upon α‐hed exposure (Figure 1C). Examination via electron microscopy highlighted deviations in the morphology of both ER and mitochondria. Specifically, cells subjected to α‐hed demonstrated pronounced ER swelling, coupled with subtle mitochondrial enlargement (Figure 1D). Previous literature has underscored the role of Alix downregulation in augmenting paraptosis,^11^, ^16^ suggesting that Alix functions as a potent indicator for paraptosis. In line with this, both protein and mRNA concentrations of Alix in HT‐29 cells treated with α‐hed were seen to reduce in a dose‐sensitive manner (Figure 1E–G).

However, α‐hed may also induce the caspase‐dependent apoptosis in CRC cells. We have conducted western blot analysis of cleaved caspase‐3 in HT‐29 cells at 24, 48 and 72 h after treatment with α‐hed (Figure S2A,B). The results showed that α‐hed activated the cleaved caspase3 expression in CRC cells. To determine if apoptosis is the decisive form of α‐hed induced cell death, we inhibited apoptosis by a caspase inhibitor, Z‐VAD fluoromethyl ketone (Z‐VAD‐FMK).^17^ Previous study has shown that 5‐FU induces caspase‐dependent apoptosis,^18^ and it could be blocked by Z‐VAD‐FMK,^19^ so we used 5‐FU as a positive control to induces apoptosis. Our study showed that both α‐hed and 5‐FU increased the expression of cleaved caspase3, however, Z‐VAD‐FMK can't rescue the elevated cleaved caspase3 expression that induced by α‐hed. In contrast, Z‐VAD‐FMK rescued the elevated cleaved caspase3 expression that induced by 5‐FU (Figure S2C,D). These results indicated that apoptosis is not the only main form of cell death following α‐hed exposure, it would support the induction of paraptosis may exert decisive influence to CRC cell death.

### 3.2 α‐Hed targets GPCRs to activate PLCβ3/IP3R/PKCα signaling pathway

**FIGURE 2:**
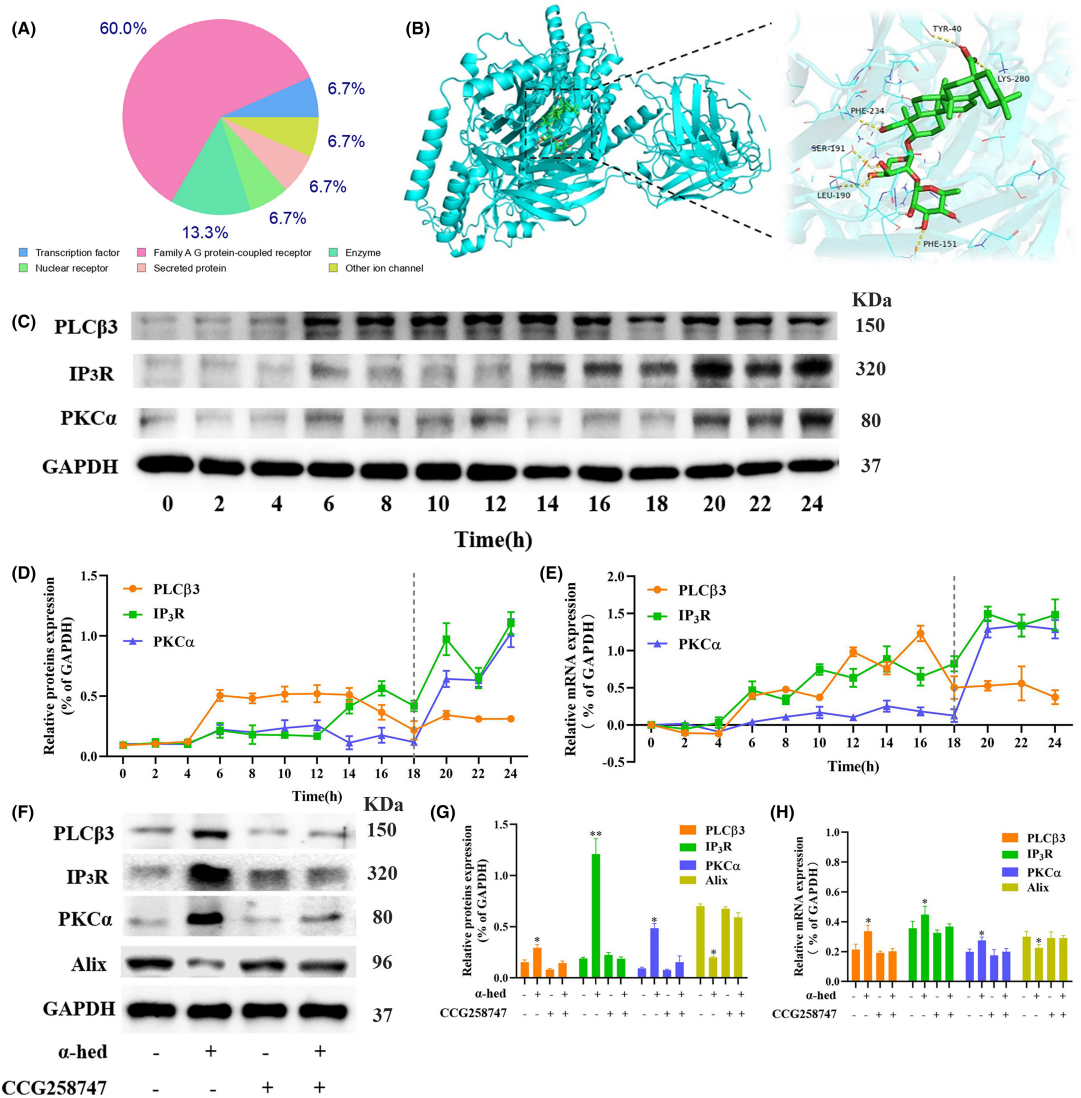
α‐Hed targets GPCRs to activate PLCβ3 /IP3R/PKCα signaling pathway. (A) Swiss Target Prediciton tool predicted 100 proteins with known ligands that are highly similar to α‐hed. (B) Molecular interaction between α‐hed and ADRA2B. (C, D) Western blot analysis of PLCβ3, IP3R, PKCα. HT‐29 cells were treated with 12 μM α‐hed for the indicated time points (0, 2, 4, 6, 8, 10, 12, 14, 16, 18, 20, 22, 24 h). GAPDH was used as loading control. ImageJ analyzed densitometric quantification of the immunoblots. (E) RT‐qPCR of PLCβ3, IP3R, PKCα. The grouping details are in consistent with above. (F, G) Western blot analysis of PLCβ3, IP3R, PKCα, Alix. HT‐29 cells were treated with α‐hed (12 μM), CCG258747 (30 nM), α‐hed combined with CCG258747 respectively for 24 h. GAPDH was used as loading control. ImageJ analyzed densitometric quantification of the immunoblots. (H) RT‐qPCR of PLCβ3, IP3R, PKCα, Alix. The grouping details are in consistent with above. *p < 0.05, **p < 0.01 compared to control group.

Swiss Target Prediciton tool predicted 100 proteins with known ligands that are highly similar to α‐hed, 60% of which are Family A GPCRs (Figure 2A), such as α2‐adrenoceptor subtypes (α2A, α2B, and α2C) (ADRA2A, ADRA2B, ADRA2C), dopamine D1 receptors (DRD1), and so forth. We then analyzed molecular simulation docking of α‐hed and GPCRs complex. For instance, α‐hed forms six hydrogen bonds with ADRA2B. Hydrogen bond is a intermolecular force that kept molecules together, the amino acids TYR‐40, LYS‐280, PHE‐234, SER‐191, LEU‐190, PHE‐151 in α‐hed forms hydrogen bonds, the molecular docking yields a binding energy of −10.2 kcal/mol (Figure 2B). Besides, other GPCRs, such as ADRA2A, ADRA2C, DRD1, and so forth, also exhibit molecular docking binding target (Figure S3). These findings are certainly suggested a direct binding between α‐hed and GPCRs.

GPCRs are links of extracellular signals to the control of cell fate, such as survival, proliferation and death.^20^ Some downstream regulators of GPCRs, such as PLCβ3, IP3R, PKCα were able to ignite Ca^2+^ signaling.^21^ CRC Cells were treated with 12 μM α‐hed for the indicated time points, at 6 h of α‐hed treatment, the protein and mRNA expression levels of PLCβ3 were markedly increased. Coincidentally, IP3R started to elevate at 6 h. At 20 h of α‐hed treatment, expression of both IP3R and PKCα were significantly increased (Figure 2C–E). CCG258747, a GPCR kinases (GRKs) subfamily–selective inhibitor, was used to impair efficacy on GPCRs‐dependent process in cells.^22^ We subsequently explored if CCG258747 could block the activation of PLCβ3/IP3/ Ca^2+^/PKCα pathway by α‐hed. CCG258747, given alone, did not significantly modify the expression of PLCβ3, IP3R, PKCα, but upregulated Alix. It significantly diminished the promoting effects of α‐hed on both the proteins and mRNA expression of PLCβ3, IP3R, PKCα while counteracted the inhibitory effect of α‐hed on Alix (Figure 2F–H), confirming the presumption that α‐hed targets GPCRs to activate PLCβ3/IP3R/PKCα signaling pathway.

### 3.3 Elevated intracellular Ca^2+^ levels are critical for α‐hed‐induced paraptosis

Cytosolic Ca^2+^ signals mediate diverse cellular responses, it generated through the coordinated translocation of Ca^2+^ across the ER membrane. ER and mitochondria are the main reservoirs of Ca^2+^.^23^ We found both ER and mitochondria swelled after α‐hed exposure, dysregulation of ion homeostasis may result in abnormal osmotic pressure, which in turn lead to morphological changes of organelles, so we presumed that α‐hed may disrupt the concentration of cytosolic Ca^2+^ level.

**FIGURE 3:**
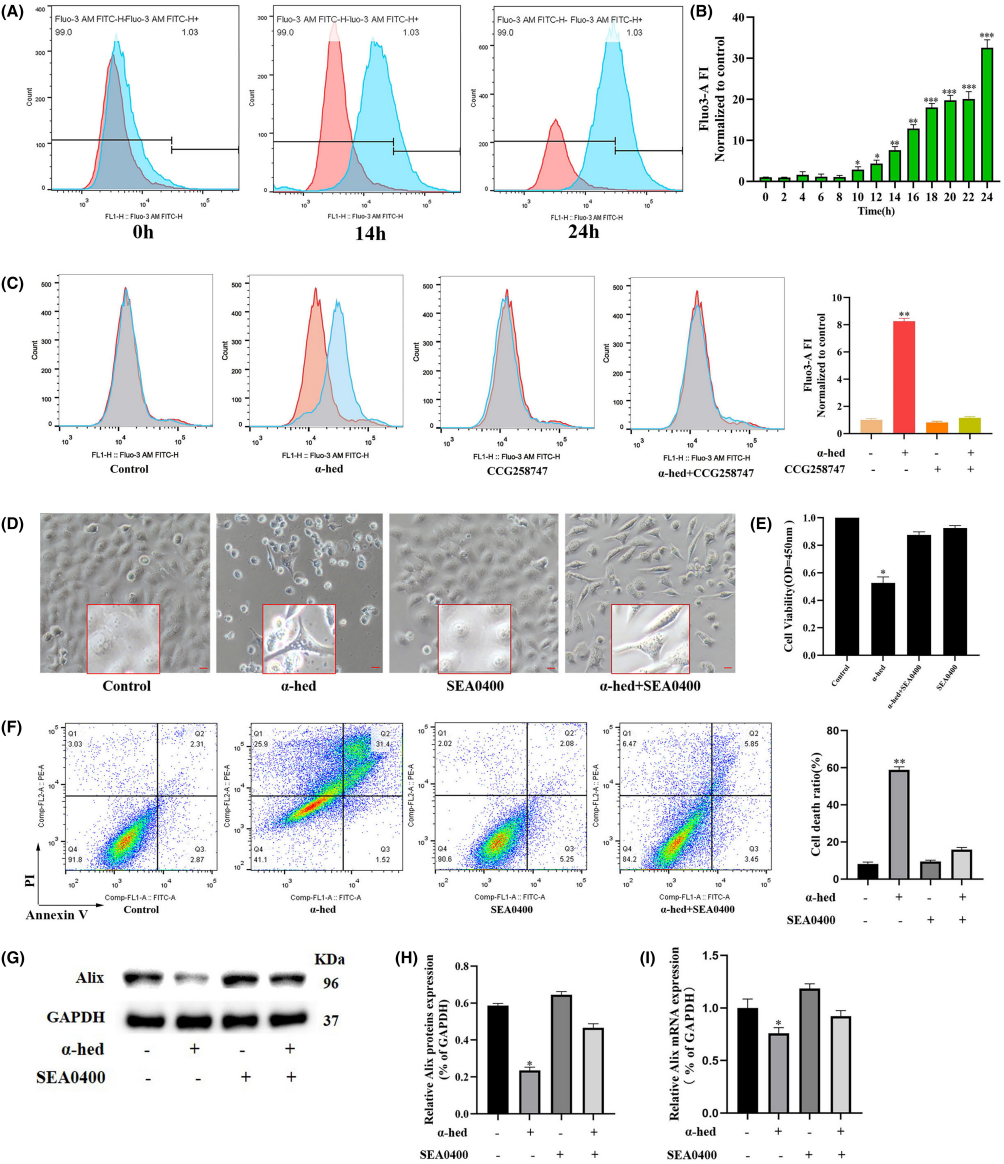
Elevated intracellular Ca^2+^ levels are critical for α‐hed‐induced paraptosis. (A, B) Fluo‐3 stained HT‐29 cells, post their exposure to 12 μM α‐hed across distinct time intervals (ranging from 0 to 24 h), underwent FACS evaluation. The graph on the left captures the fluorescence intensities (FI) for specified durations (0, 14, 24 h) of α‐hed treatment. Histogram to the right represents Fluo‐3 FI post exposure to 22 μM α‐hed across 0–24 h. (C) HT‐29 cells were treated with α‐hed (12 μM), CCG258747 (50 nM), α‐hed combined with CCG258747 respectively for 24 h, then stained with Fluo‐3 and processed for FACS analysis. FI in cells treated with α‐hed for 0, 14, 24 h were denoted in the graph. Histogram to the right represents Fluo‐3 FI in HT‐29 cells treated with α‐hed (12 μM), CCG258747 (30 nM), α‐hed combined with CCG258747 respectively for 24 h is shown. (D) HT‐29 cells were exposed to a combination of treatments, which included α‐hed (12 μM), the Na^+^/Ca^2+^ exchange inhibitor SEA0400 (50 nM), or a blend of α‐hed and SEA0400, for a period of 24 h. These were then visualized using the phase contrast microscope [scale bar =50 μm]. (E) HT‐29 Cells, as grouped in prior panels, underwent evaluation of their viability via the CCK‐8 assay. (F) HT‐29 Cells, as grouped in prior panels, was ascertained by Annexin V/PI staining method. (G, H) Western blot investigations for Alix were conducted, with cells treated as described earlier. Densitometric quantification of immunoblots was processed through ImageJ. (I) RT‐qPCR analysis of Alix was performed, with cell groupings maintained as previously detailed. Significance markers: *p < 0.05, **p < 0.01, ***p < 0.001, in relation to the control group.

Utilizing flow cytometry and the cell‐permeable Ca^2+^‐indicator dye, Fluo‐3, it was observed that when HT‐29 cells underwent treatment with α‐hed, there was a pronounced enhancement in the levels of intracellular Ca^2+^. This augmentation was noted to initiate around the 10‐h mark, escalating sharply by 14 h, and culminating in a peak at the 24‐h interval post the administration of α‐hed (Figure 3A,B). Interestingly, CCG258747 significantly diminished the promoting effects of α‐hed on Fluo‐3 fluorescence intensity (Figure 3C), confirming the presumption that α‐hed targets GPCRs to elevate intracellular Ca^2+^ levels. We then observed the localization of Ca^2+^ to the ER, mitochondria. As shown in Figure S4, the untreated HT‐29 cells exhibited evenly distributed Ca^2+^, and no obvious localization commonality with ER or mitochondria was observed. After treatment with α‐hed, the Fluo‐3 fluorescence intensity increased significantly, and aggregated in clusters, which adjacent to ER‐tracker red, coinciding with mitochondrial‐tracker‐red. These results indicate that α‐hed elevated intracellular Ca^2+^ levels, and the release of Ca^2+^ from ER is subsequently uptaken by mitochondria.

Subsequently, the research focus shifted to identifying potential evidence that suggests the role of Ca^2+^ in facilitating the paraptosis triggered by α‐hed. We tested the effect of the SEA0400, a specific Na^+^/Ca^2+^ exchange inhibitor,^24^ on α‐hed induced cytotoxicity. As shown in Figure 3D, SEA0400 effectively prevented vacuolization formation in α‐hed‐treated cells. Besides, it also blocked the inhibitory effect of α‐hed treatment in HT‐29 cells viability, while counteracted the promoting effect of α‐hed on cell death. (Figure 3E,F). Via Western blot analysis, when HT‐29 cells underwent concurrent treatment with SEA0400, the diminished Alix protein and mRNA levels caused by α‐hed were reverted to normal (Figure 3G–I).

The data gathered implies the significant elevation in intracellular Ca^2+^ levels is related to GPCRs signaling. Moreover, obstructing the Ca^2+^ pathway attenuates the paraptotic effects instigated by α‐hed in CRC cells.

### 3.4 α‐Hed activates MAPK cascade by Ca^2+^ signaling

**FIGURE 4:**
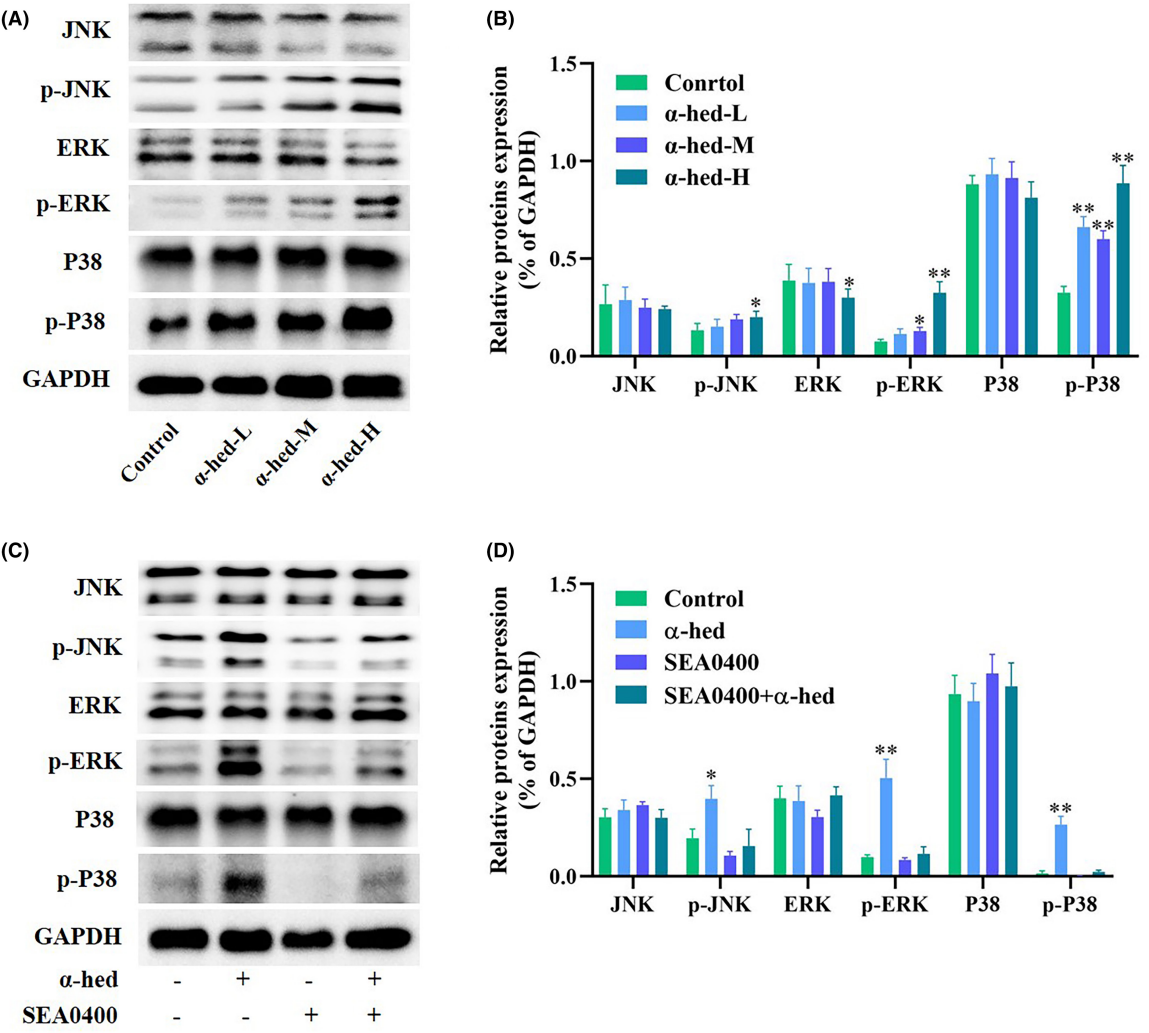
Evidence for the role of α‐hed in the initiation of the MAPK cascade via Ca^2+^ pathways. (A, B) HT‐29 cells underwent treatment with diverse concentrations of α‐hed, specifically α‐hed‐L (6 μM), α‐hed‐M (12 μM), and α‐hed‐H (24 μM), sustained over a 24 h duration. This was followed by a Western blot assessment, examining the presence and phosphorylation states of JNK, ERK, and p38. Densitometric evaluations of the obtained immunoblots were facilitated using ImageJ software. (C, D) HT‐29 cells were subjected to specific treatments: a concentration of 12 μM α‐hed, the Na^+^/Ca^2+^ exchange inhibitor SEA0400 at 50 nM, and a combined treatment involving both α‐hed (12 μM) and SEA0400 (50 nM). A subsequent Western blot evaluation focused on determining the levels and phosphorylation statuses of JNK, ERK, and p38. ImageJ was once again employed for quantifying the densitometry of the produced immunoblots. Significance indicators: *p < 0.05, **p < 0.01 when juxtaposed with the control group.

Cytosolic Ca^2+^ have been shown to trigger MAPK signaling, which has implications for paraptosis and vacuolation‐related cell demise.^25^ This study probed the phosphorylation status of JNK, ERK, and p38 to evaluate MAPK activation dynamics. Results from Western blotting revealed that while α‐hed exposure to HT‐29 cells had minimal effect on JNK, ERK, and p38 expression, there was a notable augmentation in the phosphorylation of MAPK proteins, demonstrating a dose‐responsive relationship (Figure 4A,B). Moreover, concurrent treatment with SEA0400 in HT‐29 cells thwarted the α‐hed‐triggered phosphorylation states of JNK, ERK, and p38 (Figure 4C,D). Such findings substantiate the crucial role of the MAPK signaling cascade in the Ca^2+^ driven paraptosis in CRC cells.

### 3.5 Inhibition of chemo‐resistant CRC cell line by α‐hed may be effective compared to chemotherapeutic agents

**FIGURE 5:**
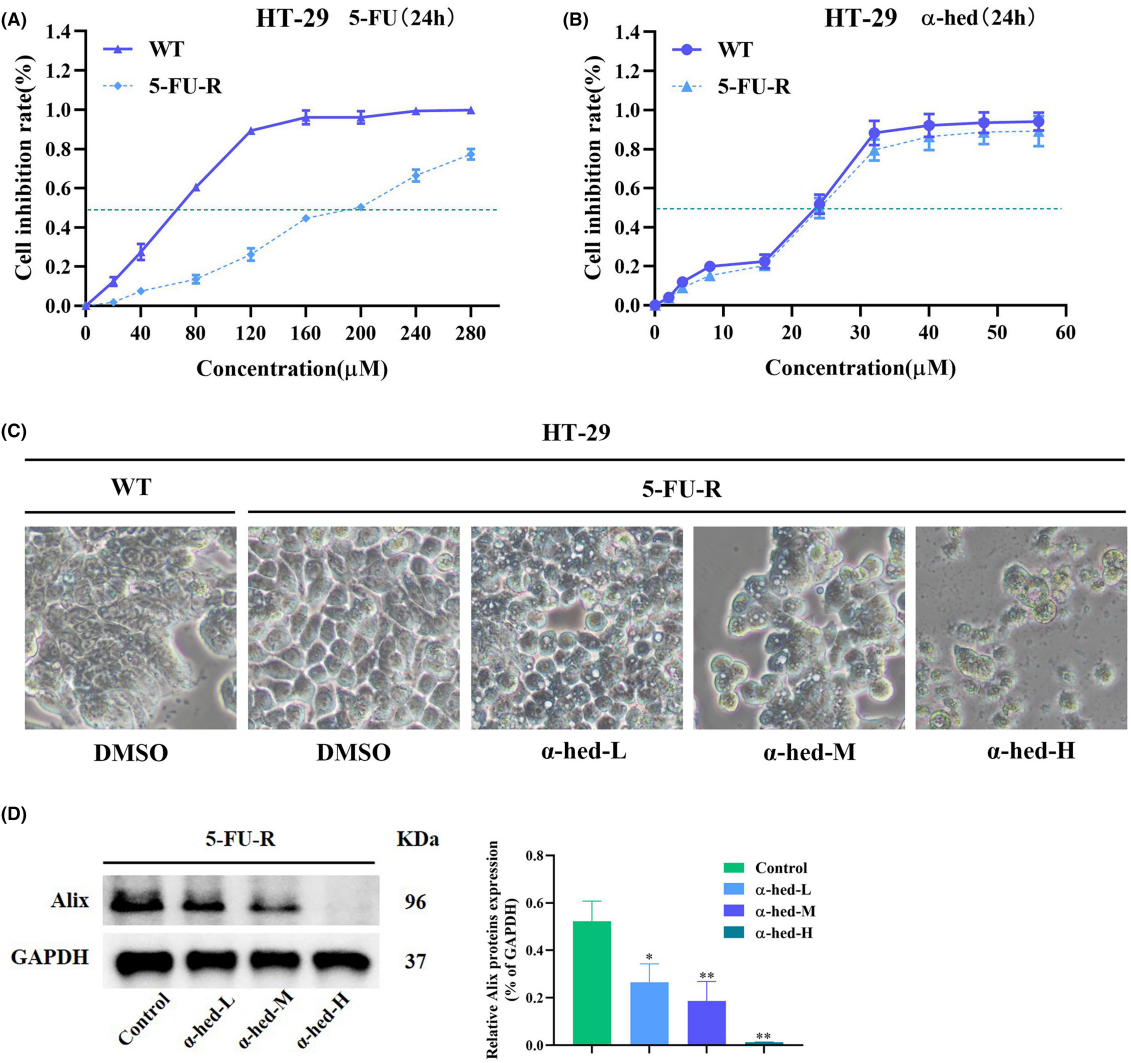
α‐Hed overcomes chemotherapy resistance in CRC cells by induction of paraptosis. (A, B) Effects of 5‐FU and α‐hed on HT‐29 cell viability. Cells were developed resistant to 5‐FU through a dose‐escalation assay. Parental cells (WT) and 5‐FU‐resistant lines (5‐FU‐R) were then exposed to increasing concentrations of 5‐FU (0, 40, 80, 120, 160, 200, 240, 280 μM) or α‐hed (0, 2, 4, 8, 16, 24, 32, 40, 48, 56 μM) for 24 h. (C) Phase images of 5‐FU‐R HT‐29 cells were exposed to α‐hed‐L (6 μM), α‐hed‐M (12 μM), α‐hed‐H (24 μM) for 24 h [scale bar =20 μm]. (D) Western blot analysis of Alix. The grouping details are in consistent with above. ImageJ analyzed densitometric quantification of the immunoblots. *p < 0.05, **p < 0.01 compared to control group.

Most chemotherapeutic agents, such as 5‐FU, exert inhibitory activity in cancer by inducing apoptosis, thus non‐apoptotic cell death may suggest strategies that could offer alternatives to chemo‐resistant CRC therapeutic approaches. We then determined whether α‐hed, which induces paraptosis, is able to target resistant CRC cancer cells. To test this hypothesis, we set up 5‐FU‐resistant CRC clones by a step‐wise dose escalation study as reported previously.^26^ Cell viability in the presence of 5‐FU clearly showed that derived HT‐29 clones are resistant to 5‐FU, the IC50 of 5‐FU‐resistant and parental HT‐29 clones are being at approximately 200, 75 μM respectively (Figure 5A). Remarkably, reduction of viability by exposure of α‐hed in 5‐FU‐resistant and parental HT‐29 cells showed barely difference (Figure 5B). α‐hed also induced cytoplasmic vacuolation in 5‐FU resistant HT‐29 cells, indicating initiation of paraptosis‐like features (Figure 5C). Besides, α‐hed also decreased expression of Alix in 5‐FU‐resistant HT‐29 cells in a does‐dependent manner (Figure 5D). Collectively, α‐hed may effectively inhibit apoptosis‐based 5‐FU‐resistant CRC cells via inducing paraptosis‐like cell death.

### 3.6 α‐Hed inhibits 5‐FU‐R HT‐29 xenograft growth in mice

In order to validate whether α‐hed possesses inhibitory effects on chemo‐resistant CRC in vivo, a study was conducted in nude mice with chemo‐resistant HT‐29 tumor xenografts to assess the therapeutic potential.

**FIGURE 6:**
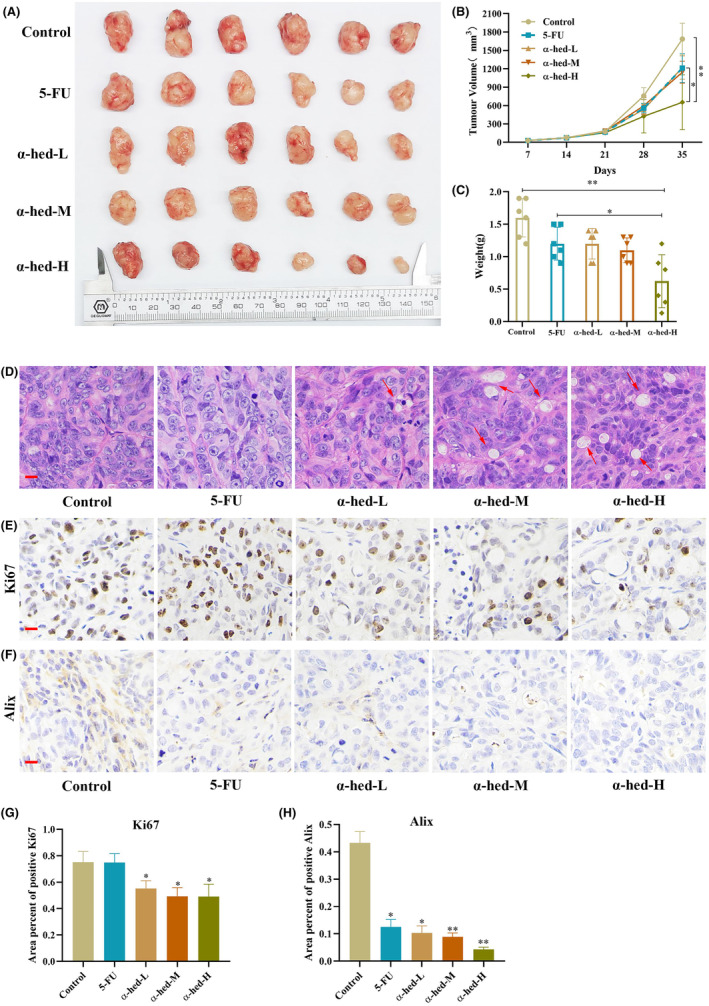
α‐Hed inhibits 5‐FU‐R HT‐29 xenograft growth in mice. (A) Visual documentation of excised tumor tissues obtained from the experimental mice. (B) Tracing the progression of tumor dimensions at specific intervals after the introduction of HT‐29 cells into the nude mice. (C) The final evaluation of tumor mass at the termination of the experimental period. (D) Histological examination via H&E stain of the collected tumor samples. Noticeable α‐hed‐triggered cytoplasmic vacuolation is highlighted with red pointers [scale bar = 100 μm]. (E–H) IHC analysis of the tumor sections, concentrating on Ki‐67 and Alix expression. The presence of immunoreactive substances was identified using the DAB chromogen, appearing in a brown hue [scale bar = 100 μm].

These mice underwent treatment via intraperitoneal injection of either 5‐FU (25 mg/kg) or varying dosages of α‐hed (0.5, 1, 1.5 mg/kg). Observations indicated that 5‐FU administration barely led to a suppression in CRC progression relative to the control. Furthermore, intermediate α‐hed doses (0.5, 1 mg/kg) exhibited marginal tumor growth deceleration. The dose of 1.5 mg/kg of α‐hed marked a significant decline in tumor volumes and weights (Figure 6A–C), which seemed to surpass the inhibitory capability of 5‐FU. Analysis of excised tumor samples further elucidated α‐hed's impact on CRC expansion. Histological evaluation via H&E staining displayed cytoplasmic vacuolation, mirroring attributes of paraptosis‐resembling cellular fatality (Figure 6D). Yet, it remains imperative to highlight observed fluctuations in body weight measurements across both 5‐FU and α‐hed administered groups (Figure S5).

Through IHC staining, it became evident that administering α‐hed led to a notable suppression in cellular proliferation, as illustrated by the diminished expression of Ki67. Moreover, signs pointing towards paraptosis as the decreased expression of Alix (Figure 6E–H). In contrast, the PLCβ3/IP3R/PKCα pathway was increased (Figure S6). Additionally, cell death was witnessed (Figure S7). All these observations collectively hint that α‐hed induces paraptosis and contributes to an observable restraint on chemo‐resistant CRC growth when assessed in vivo.

## 4 DISCUSSION

Since cancer cells develop adaptive abilities to escape apoptosis, other forms of programmed cell death has emerged to overcome apoptotic‐based drug resistance.^27^ Accumulating evidences have shown that compounds from natural plants has been an efficient alternative way in inventing anti‐cancer drugs.^28^ In the current study, a natural plant substance α‐hed with pronounced cytotoxic efficacies to CRC cells was identified, it triggers paraptosis, instead of only apoptotic cell death in CRC cells. Here we show that α‐hed directly targeting multiple subunits of GPCRs to stimulate Ca^2+^ release from ER stores, and subsequently activate the characteristic pathway of paraptosis, the MAPK cascade. Extensive cytoplasmic vacuolations are strongly associated with elevated intracellular Ca^2+^ levels, osmotic pressure that induced by dysregulation of ion homeostasis may in turn lead to ER/mitochondrial dilation. Besides, the Na^+^/Ca^2+^ exchange inhibitor, SEA0400 effectively prevented vacuolization formation in α‐hed‐treated cells. Interestingly, Ca^2+^ signaling has been highlighted to be detrimental to cancer cells,^29^ and we found SEA0400 effectively block the cytotoxic effect of α‐hed treatment in HT‐29 cells. As we presumed, α‐hed was effective against chemotherapy‐resistant CRC both in vivo and in vitro. These findings suggest that α‐hed is a potential therapeutic candidate for CRC via a Ca^2+^‐mediated paraptotic mechanism, the central players behind the anti‐cancer activity were GPCRs‐mediated downstream pathways.

**FIGURE 7:**
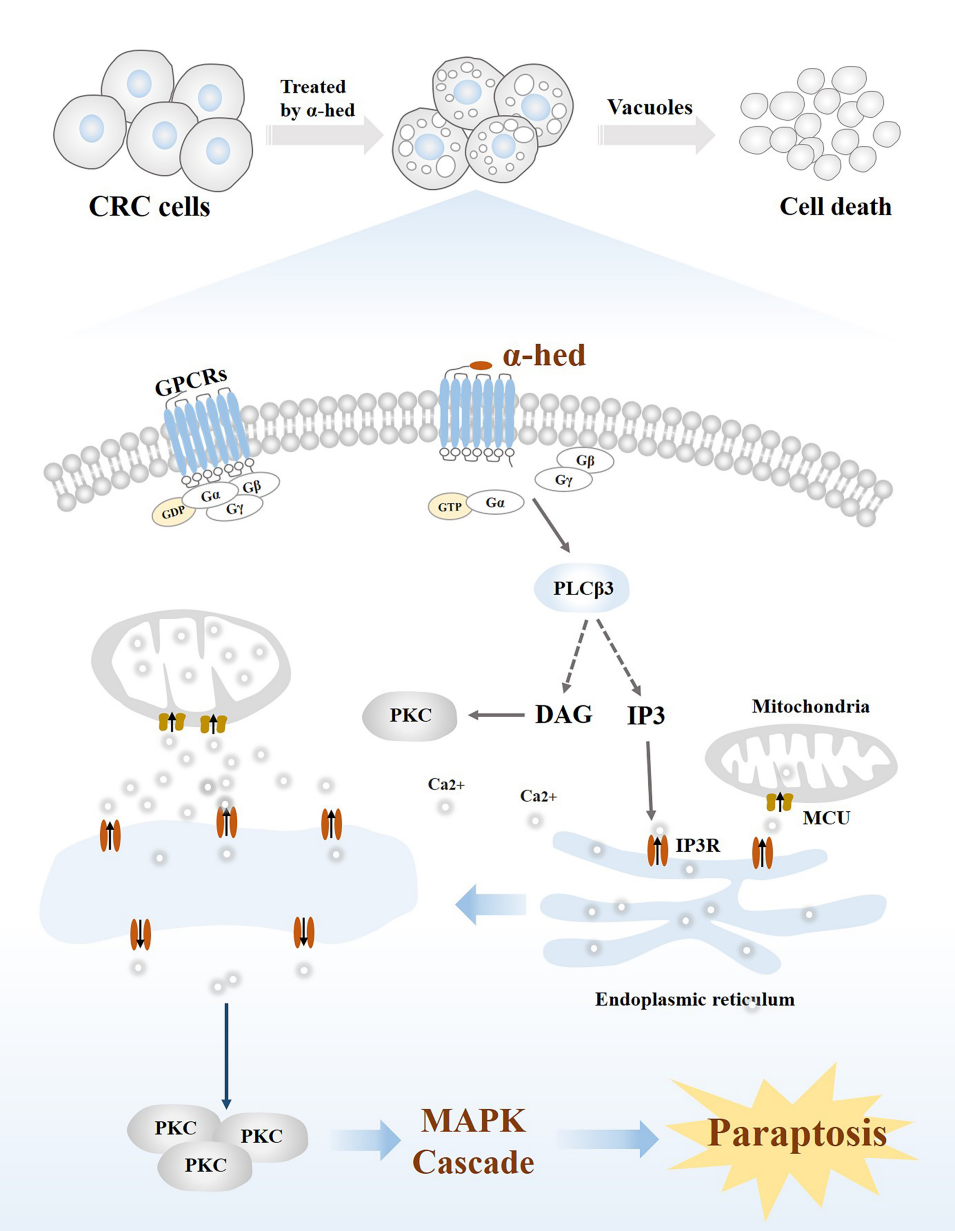
A schematic mechanism underlying α‐hederin induces calcium‐mediated paraptosis‐like cell death by targeting GPCRs in colorectal cancer (CRC).

GPCRs activation is universally fundamental for the regulation of cell functions.^30^, ^31^ The GPCRs intracellular domain is bound to a heterotrimer of guanine nucleotide‐binding proteins, comprising a GDP‐bound Gα, Gβ and Gγ subunits.^32^ Upon ligand binding, replacement of GDP with GTP, induces the dissociation of the heterotrimer, as well as the detachment of Gα from the Gβγ subunits. The liberated Gα and Gβγ dimers then evoke signaling pathways that mediate multiple cellular functions.^33^, ^34^ Gα binds to and activates PLCβ enzymes, which hydrolyses the membrane lipid phosphatidylinositol‐4,5‐bisphosphate (PIP2) into the IP3 and DAG, and this is ultimately followed by a transient rise in Ca^2+^ level via release from ER stores.^35^ Four PLCβ isozymes (PLCβ1‐4) share the same hydrolytic reaction,^36^ of which PLCβ3 is elevated by α‐hed, and we also demonstrated its elevated protein expression after α‐hed exposure. PKC isozymes (α, β, γ) is a group of serine/threonine protein kinases that are physiologically activated by DAG and Ca^2+^,^37^ it regulates cell death by transmitting extracellular signals to downstream pathways, MAPK is the one of the responding cascades.^38^ We then found protein expression of PKCα could be elevated after α‐hed exposure, but counteracted by CCG258747. Of note, we also found MAPK is activated by α‐hed, as the phosphorylated forms of ERK, p38, and JNK were all increased, and this effect could be diminished by SEA0400, supporting the presumption that α‐hed targets GPCRs to induce Ca^2+^ mediated paraptosis‐like cell death in CRC (Figure 7).

Therefore, α‐hed may render an alternative option to overcome apoptotic‐based drug resistance by inducing paraptosis, and exploiting an opportunity for natural anti‐cancer drug development.

## 5 CONCLUSION

α‐Hed promotes paraptosis‐like cell death in CRC. The mechanism is facilitated by the surge in GPCRs mediated Ca^2+^ signaling, which subsequently triggering MAPK cascade. Comprehensive evaluations further corroborate the efficacy of α‐hed in chemo‐resistant CRC both in vivo and in vitro conditions. Data from this research hints at the possibility of considering α‐hed as a potential alternative approach in CRC therapy.

## AUTHOR CONTRIBUTIONS

**Xiwu Rao:** Conceptualization (equal); data curation (equal); formal analysis (equal); funding acquisition (equal). **Ziwen Li:** Data curation (equal); resources (equal). **Qinchang Zhang:** Project administration (equal); validation (equal); visualization (equal). **Yueyang Lai:** Visualization (equal); writing – original draft (equal). **Jianrong Liu:** Resources (equal). **Liu Li:** Methodology (equal); writing – review and editing (equal). **Haibo Cheng:** Project administration (equal); resources (equal); writing – review and editing (equal). **Weixing Shen:** Resources (equal); supervision (equal); validation (equal). **Dongdong Sun:** Project administration (equal); supervision (equal); validation (equal); writing – review and editing (equal).

## FUNDING INFORMATION

This work was supported by grants from the National Natural Science Foundation of China (no. 82374287, 82305340, 81973523, 82074318), Key projects of Traditional Chinese Medicine Technology Development Plan of Jiangsu Province (no. ZD202201), The Science and Technology Development Fund, Macau SAR0098/2021/A20048/2023/AFJ, China Postdoctoral Science foundation (no. 2023M740860).

## ETHICS STATEMENT

The experiments using the animals were approved by the Institutional and Local Committee on the Care and Use of Animals of Nanjing University of Chinese Medicine (Ethical approval number, 202110A025).

## Supporting information


Data S1:

## Data Availability

All datasets and materials supporting the conclusion for this study are included in the article.
