# Supplementary material for: α‐Hederin induces paraptosis by targeting GPCRs to activate Ca2+/MAPK signaling pathway in colorectal cancer
Source: Cancer Med. 2024 Apr 25;13(8):e7202. doi: 10.1002/cam4.7202 (PMC11043672; doi:10.1002/cam4.7202)
Supplement: Supplementary file 1 — Data S1: [file CAM4-13-e7202-s001.docx]

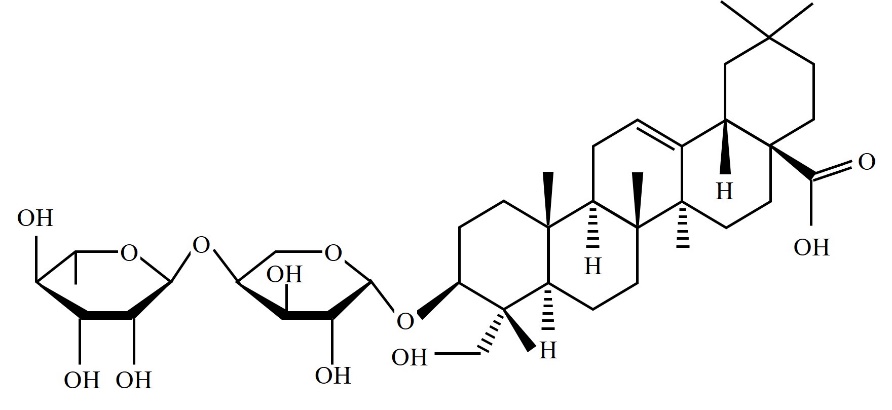


Fig.S1. The chemical structure of α-hederin.


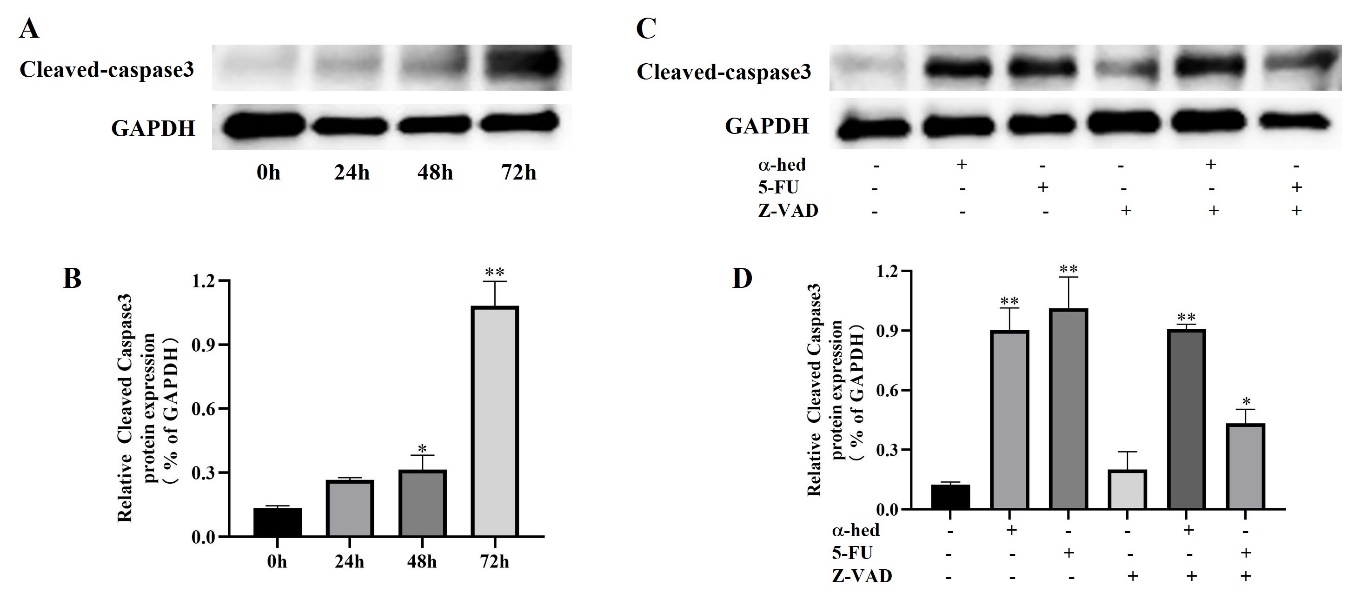


Fig.S2. Western blot analysis of Cleaved caspase3. (A-B) HT-29 cells were treated with α-hed 12µM for 0h, 24 h, 48h, 72h respectively. (C-D) HT-29 cells were treated with α-hed (24 μM), 5-FU (100 μM), Z-VAD-FMK (50 μM), 5-FU combined with Z-VAD-FMK, α-hed combined with Z-VAD-FMK respectively for 24h.ImageJ analyzed densitometric quantification of the immunoblots. **P* <0.05, ***P* < 0.01 compared to control group.


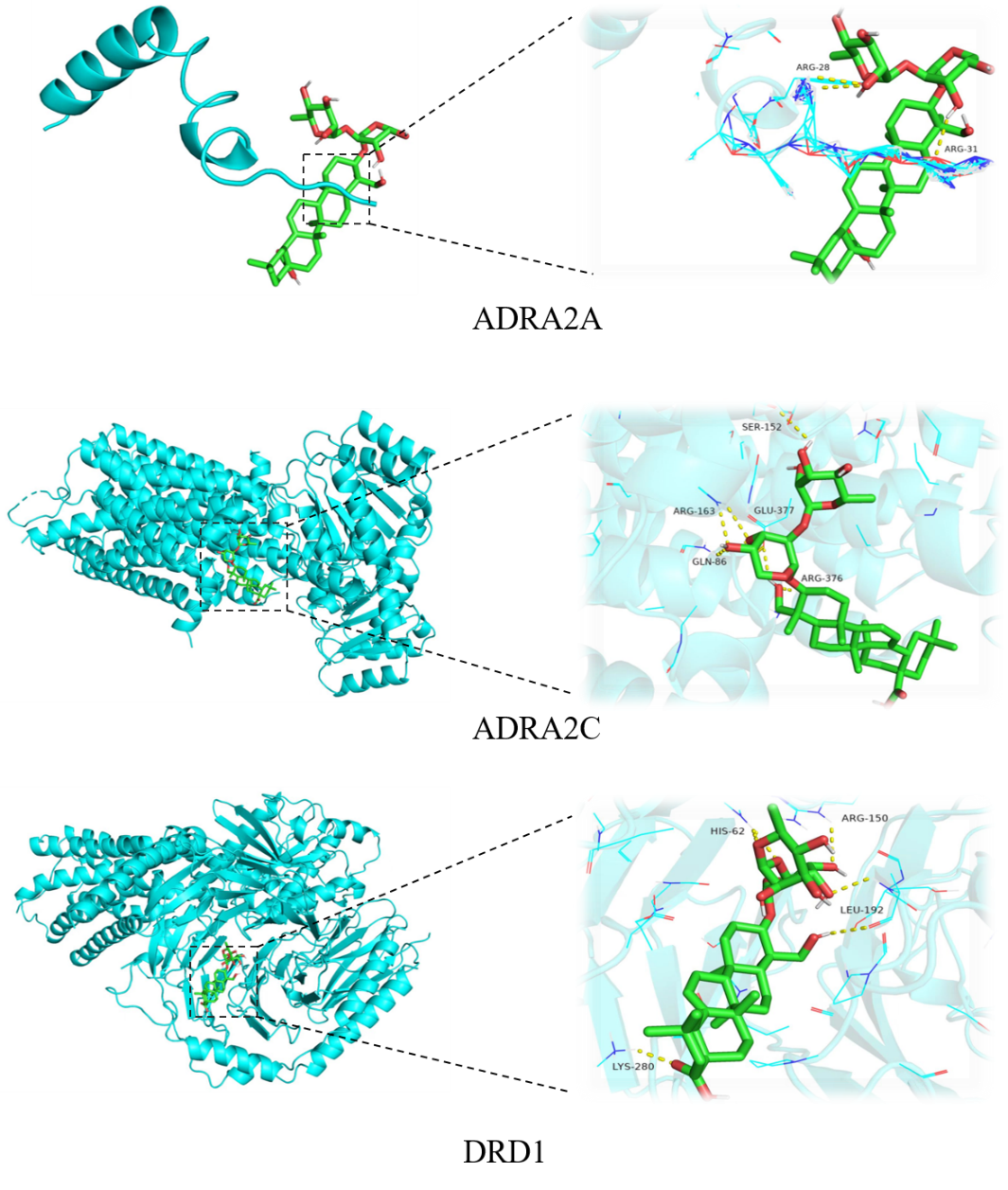


Fig.S3. Molecular interaction between α-hed and ADRA2A, ADRA2C, DRD1.


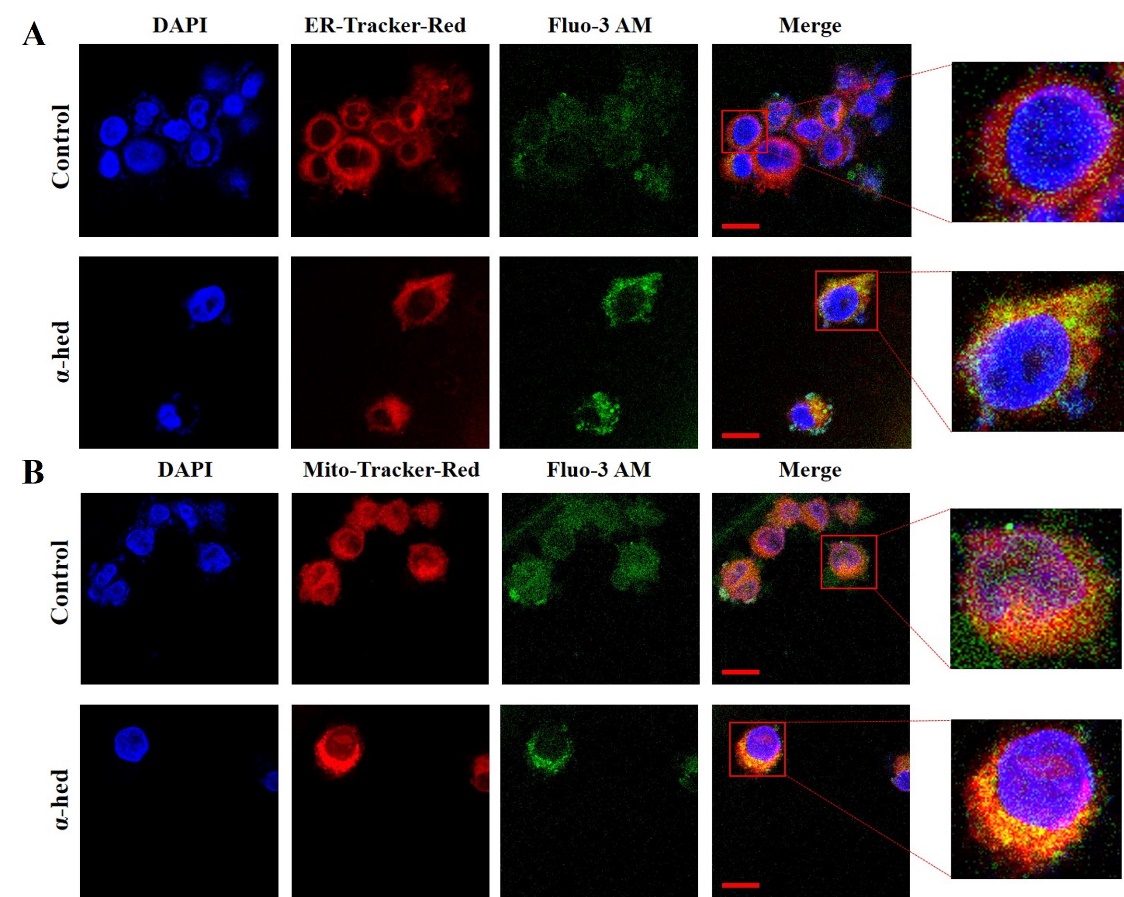


Fig.S4. Co-localization of Ca^2+^ and ER/mitochondrion after α-hederin treatment. (A) HT-29 cells treated with or without 12 μM α-hed for 24 h were co-stained with Fluo-3 and ER-Tracker Red, then observed under the Confocal laser scanning microscope [scale bar =50 μm]. (B) HT-29 cells treated with or without 12 μM α-hed for 24 h were co-stained with Fluo-3 and Mito-Tracker Red, then observed under the Confocal laser scanning microscope [scale bar =50 μm].


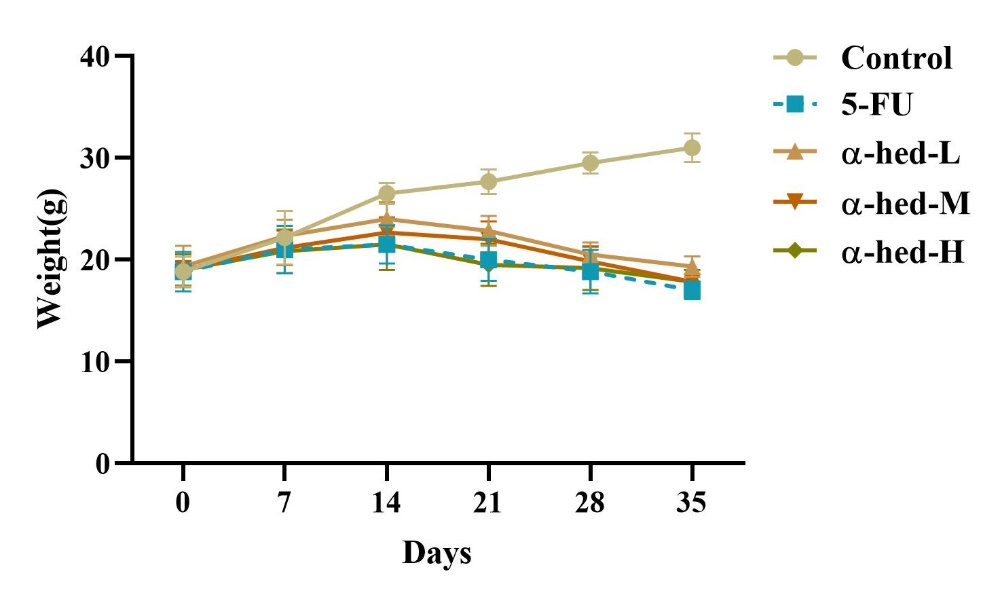


Fig.S5. The body weight of mice was measured weekly.


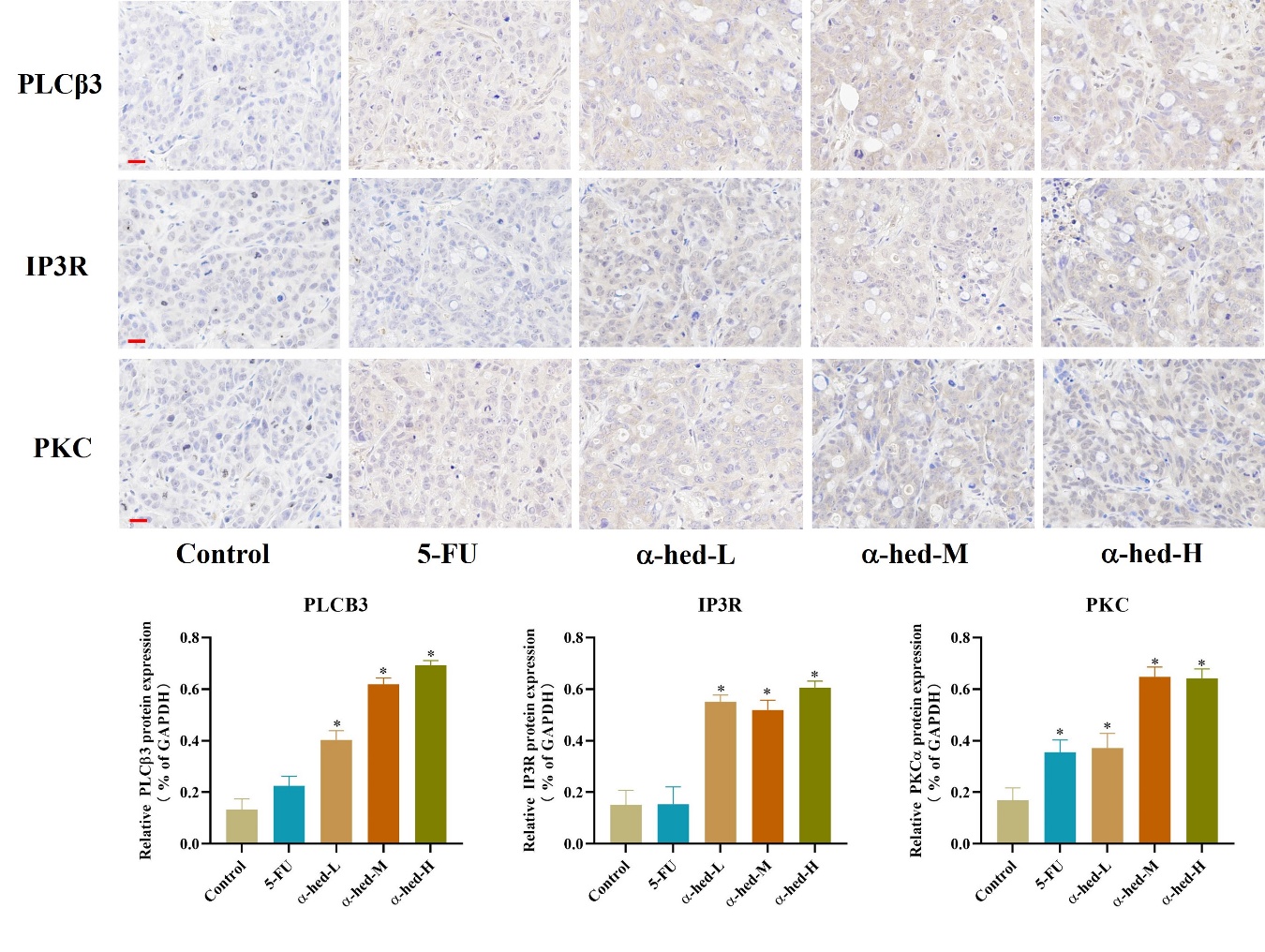
 Fig.S6. IHC staining of PLCβ3/IP_3_R/PKCα pathway. [scale bar =100 μm].


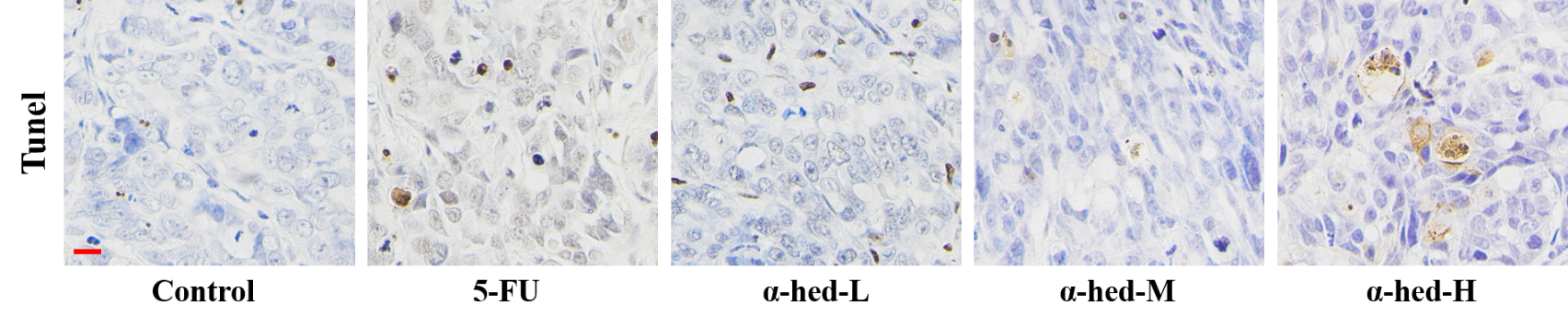


Fig.S7. TUNEL staining of tumor specimens. [scale bar =100 μm].
